# Pharmacogenetics and adhd treatment outcomes in the danish population-based IPSYCH sample

**DOI:** 10.1192/j.eurpsy.2021.395

**Published:** 2021-08-13

**Authors:** C. Lunenburg, K. Ishtiak-Ahmed, J. Thirstrup, C. Gasse

**Affiliations:** 1 Department Of Clinical Medicine, Aarhus University, Aarhus, Denmark; 2 Dep. Affective Disorders, Aarhus University Hospital Psychiatry, Aarhus N, Denmark; 3 Department Of Biomedicine, Faculty of Health, Aarhus University, Aarhus, Denmark; 4 Psychosis Research Unit, Aarhus University Hospital Psychiatry, Aarhus N, Denmark; 5 Centre For Integrated Register-based Research, Aarhus University, Aarhus, Denmark

**Keywords:** Pharmacogenetics, ADHD, atomoxetine

## Abstract

**Introduction:**

Pharmacogenetics (PGx) studies genetic variance and related differences in drug outcomes. The aim of PGx testing is to increase therapy efficacy and safety, by applying e.g. dose adjustments in patients with a specific geno- or phenotype. PGx guidelines for psychotropic drugs are available (PGx drugs), including atomoxetine used in the treatment of attention deficit hyperactivity disorder (ADHD). In Denmark, broad implementation of PGx is currently still low, possibly due to the lack of population-based studies investigating the real-world impact of PGx variability.

**Objectives:**

The aim of this study is to investigate the association of PGx variability (patients’ genotype/phenotype) in users of atomoxetine and different treatment outcomes in a large population-based sample of individuals with ADHD.

**Methods:**

This study will use data of the large Danish population-based iPSYCH case-cohort study sample including information on genetic variants, prescription drug use and outcome data, e.g. psychiatric and somatic hospitalizations and death. The study population comprises all individuals diagnosed with ADHD born 1981-2005 with at least one prescription for atomoxetine between 1995 and 2016. All individuals will be categorized according to their CYP2D6 phenotypes. We will perform Cox regression analysis to estimate the hazard ratios comparing the rates of the different outcomes in people with different phenotypes adjusted for a number of confounding factors.

**Results:**

We have identified approximately 20,000 individuals with ADHD, of whom an estimated 10-20% have filled at least one prescription of atomoxetine.

**Conclusions:**

We expect results in the beginning of 2021.

**Disclosure:**

We thank the iPSYCH consortium, in specific the iPSYCH PI’s (Merete Nordentoft, Anders Børglum, Preben B. Mortensen, Ole Mors, Thomas Werge and David M. Hougaard). The iPSYCH project is funded by the Lundbeck Foundation Denmark and the universities and un

